# Diseases of the Fingers

**Published:** 1891-09-19

**Authors:** 


### DISEASES OF THE FINGERS (*concluded*).

#### G.—The Skin, &c.

Webbed Fingers ("Syndactylism") may be either traumatic (from burns, &c.) or congenital. The latter condition sometimes includes all the fingers and thumb, but more usually it is confined to two or three only. Most commonly it is the middle and ring fingers that are joined together, the connecting skin extending to the extreme tips or being only a slight exaggeration of the natural inter-digital fold. Dr. Goldmann (of Freiburg) has recently pointed out that these cases must be considered as arrested development. The hand is formed at first as a rounded, flattened bud springing from the side of the body. This is followed by a growth from behind, which thrusts the first forwards and becomes the arm. Then the fingers appear as small projections, caused by atrophy of the inter-digital tissue. If this should remain instead of undergoing absorption the result is "webbed fingers." The thumb is the first to separate from the undifferentiated mass, hence it is very rare to have this included in the deformity. It should be noticed that, although the separation into digits occurs very early, it is preceded by changes in the deeper parts, such as formation of distinct cartilaginous phalanges, &c.

The condition is often hereditary, and may possibly be looked upon as a reversion to the webbed fingers and toes of some remote aquatic ancestor. The *treatment* consists in freeing the conjoined parts, and this may be effectually carried out in many ways, but most readily in one of the three following: (1) By merely dividing the web with a scalpel, and allowing the raw surfaces to granulate up. It is necessary to keep a piece of lint between the fingers until the healing process is complete to prevent re-union. This plan succeeds perfectly in most cases. (2) By a V-shaped incision on the dorsal surface of the abnormal hand, with the apex at the distal portion. This is reflected back, and, when the fingers are separated, is folded over and stitched to the raw edges at the sides. (3) By the operation introduced by Didot (and described in most surgery books) of making a flap from the front of one finger and another from the back of the adjoining one. The flaps so made are sutured on to the raw surfaces. Besides these methods, compression of the skin by a long bladed forceps until it sloughs through and ligature with wire have been practised. These, however, are painful and unsatisfactory.

Congenital Hypertrophy of one or more fingers is sometimes found. The skin, cellular tissue, and fat are affected. This must not be confounded with the peculiar disease called "Acromegaly," in which the gradual enlargement involves other parts as well, is seated in the bones, and is not found in the young. A very marked case of the congenital condition is reported and figured by Mr. T. F. Raven in the *Illustrated Medical News* for January 5th, 1889. This occurred in a girl, and the right index finger and thumb were much larger than the left. Nothing can be done, surgically, in either case.

Eruptions occur on the fingers as elsewhere. Eczema is excited in this position by constantly handling fine powders (as in bakers, grocers, etc., where flour seems to be the irritating cause) and by soda (as in washerwomen). The oleate of zinc in ointment generally effects a cure. Any dermatitis in the neighbourhood of an inter-phalangeal joint may involve the fibrous tissues, and even the ligaments, secondarily. Besnier has described thickening of the joints in psoriasis which may be of this nature.

Œdema of the hands and fingers is sometimes seen without any obvious cause. It occurs in the dorsum of the hand most commonly, or at the bases of the fingers in middle-aged women. The pathology is obscure, but it is probably due to a localised dilatation of the blood vessels. It generally lasts only a day or two. So many conditions (such as sprains of the wrist, blows, bites by insects, etc.) may give rise to a similar state that it is a little doubtful if the reported cases of this localized œdema can be considered as sufficient evidence of a distinct disease.

Supernumerary Fingers ("Polydactylism").—Voight has recorded a case in which thirteen fingers existed on one hand; seven or eight have been described more frequently, but even this number is extremely rare. The commonest form is an extra thumb, which may have a joint at its base, or the phalanx may be bifid and the appendage firmly fixed. Next to this in point of frequency is a little finger growing from the base of the normal one. These conditions are markedly hereditary; thus, Mr. Johnson reported (in the *American Medical Times* for 1860) a family which possessed these extra members for four generations. In this, as in other instances, the peculiarity was transmitted through the mother; the males, although they had the deformity, do not seem to have transmitted it to their children. The treatment is simple; the extra finger should be removed by making small flaps, and, if necessary, using the bone forceps. It is important to operate early; in fact, the younger the better. In the case of supernumerary thumbs and little fingers, the part may be snipped off in a baby without giving an anæsthetic, if done rapidly. Nothing is gained by delay.

In conclusion, we would again point out that the correct understanding of diseases of the fingers depends on a knowledge of the anatomy and functions of the parts concerned. Points which at first seem trifling, such as the exact method of the insertion of the tendons, the structure of the nail and its bed, the varying thickness of the skin, the peculiarities of the arterial supply, and the different kinds of fascia—all these details are most important. This holds good, too, of treatment; when the nature of the tissues is understood it is easy to see how prompt and energetic the surgeon must be in dealing with inflammatory affections, and how delay may cause the most serious consequences.

